# Selective Digestive Decontamination: A Comprehensive Approach to Reducing Nosocomial Infections and Antimicrobial Resistance in the ICU

**DOI:** 10.3390/jcm13216482

**Published:** 2024-10-29

**Authors:** María Martínez-Pérez, Rosario Fernández-Fernández, Rocío Morón, María Teresa Nieto-Sánchez, María Eugenia Yuste, Xando Díaz-Villamarín, Emilio Fernández-Varón, Alberto Vázquez-Blanquiño, Ana Alberola-Romano, José Cabeza-Barrera, Manuel Colmenero

**Affiliations:** 1 Hospital Pharmacy, Hospital Universitario San Cecilio, 18016 Granada, Spain; maria.martinez.p.sspa@juntadeandalucia.es (M.M.-P.); mariat.nieto.sanchez.sspa@juntadeandalucia.es (M.T.N.-S.); xandodv@ugr.es (X.D.-V.); jose.cabeza.sspa@juntadeandalucia.es (J.C.-B.); 2 Critical Care Department, Hospital Universitario San Cecilio, 18016 Granada, Spain; rosario.fernandez.f.sspa@juntadeandalucia.es (R.F.-F.); mariae.yuste.sspa@juntadeandalucia.es (M.E.Y.); manuel.colmenero.sspa@juntadeandalucia.es (M.C.); 3 Instituto de Investigación Biosanitaria de Granada (Ibs.Granada), 18012 Granada, Spain; emiliofv@ugr.es (E.F.-V.); alberto.vazquez.sspa@juntadeandalucia.es (A.V.-B.); ana.alberola.sspa@juntadeandalucia.es (A.A.-R.); 4 Department of Pharmacology, Center for Biomedical Research (CIBM), University of Granada, 18016 Granada, Spain; 5 Clinical Microbiology Service, Hospital Universitario San Cecilio, 18016 Granada, Spain

**Keywords:** selective digestive decontamination, antibiotic consumption, MDR bacteria, ICU

## Abstract

**Background/Objective:** Multidrug-resistant (MDR) bacteria pose a significant threat to global health, especially in intensive care units (ICUs), where high antibiotic consumption drives antimicrobial resistance. Selective digestive decontamination (SDD) is a strategy designed to prevent nosocomial infections and colonization by MDR pathogens. This study aimed to evaluate the impact of implementing an SDD protocol on antibiotic consumption and colonization by carbapenemase-producing Enterobacterale (CPE) in a specific ICU setting. **Methods:** This quasi-experimental study was conducted in the ICU of a university hospital from June 2021 to June 2023. Patients were divided into two groups: pre-intervention (before SDD) and post-intervention (after SDD implementation). Data on antibiotic consumption (expressed as defined daily doses (DDDs) per 100 stays), nosocomial infections, colonization rates, and the incidence of MDR bacteria were collected. A statistical analysis was conducted to compare the pre- and post-intervention groups. **Results:** A total of 3266 patients were included, with 1532 in the pre-intervention group and 1734 in the post-intervention group. The implementation of the SDD protocol resulted in a significant reduction in total antibiotic consumption (*p* = 0.028), with notable decreases in carbapenem use (*p* < 0.01) and colonization by CPE (*p* = 0.0099). The incidence of nosocomial infections also decreased in the post-SDD group, although this reduction was not statistically significant. **Conclusions:** The implementation of the SDD protocol in this ICU setting significantly reduced antibiotic consumption and colonization by CPE. These findings suggest that SDD may be a valuable tool in managing antimicrobial resistance in critical care settings, without contributing to the development of MDR bacteria.

## 1. Introduction

Antimicrobial resistance (AMR) is identified by the World Health Organization (WHO) as one of the top ten global public health threats. This resistance is exacerbated by the indiscriminate and inappropriate use of antibiotics, leading to the emergence of multidrug-resistant (MDR) or pan-resistant bacteria, often referred to as ‘superbugs’. These bacteria cause common infections that cannot be treated with conventional antibiotics [1].

The incidence of MDR microorganisms is rising in both community and hospital settings. The emergence of MDR bacteria in areas with high antibiotic consumption, such as intensive care units (ICUs), increases the population’s vulnerability to nosocomial infections, posing a serious public health risk [2]. Infections caused by MDR bacteria are associated with therapeutic failure, prolonged hospital stays, increased healthcare costs, and higher mortality rates, particularly for critically ill patients [3]. The societal costs of antibiotic resistance are estimated at EUR 1.5 billion, with AMR being linked to approximately 25,000 deaths annually in the European Union [4].

The prevention of, early diagnosis of, and appropriate treatment for nosocomial infections are among the greatest challenges in inpatient care [5,6,7,8]. According to the ENVIN-HELICS (National Surveillance Study of Nosocomial Infection in ICU) report, it is essential to reduce the duration of antibiotic treatment for hospital-acquired infections, as patients often receive prolonged antibiotic therapy [9].

**Figure 1:**
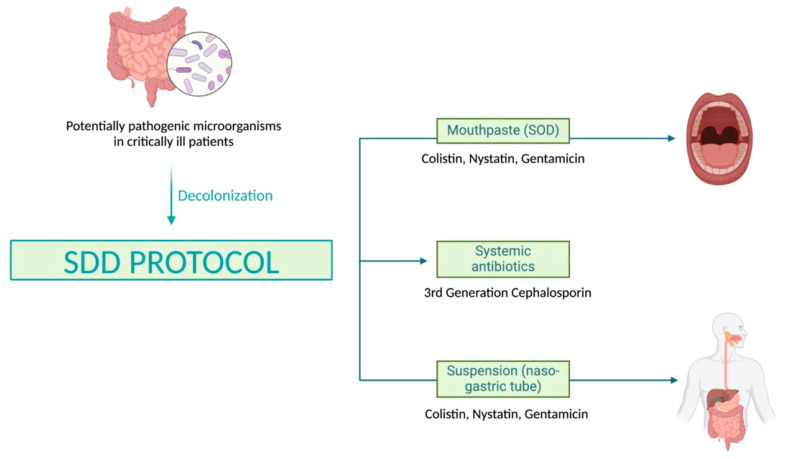
Design of the selective digestive decontamination (SDD) protocol. SOD: selective oropharyngeal decontamination. Treatment consisted of a combination of a mouthpaste (SOD) and a suspension administered through a nasogastric tube, along with a systemic parenteral antibiotic. The mouthpaste and suspension were composed of a mixture of two antibiotics (gentamicin and colistin) and an antifungal agent (nystatin).

In this context, selective digestive decontamination (SDD) is a strategy used in ICUs to prevent infections, particularly ventilator-associated pneumonia (VAP) [10]. SDD involves applying an oropharyngeal paste (selective oropharyngeal decontamination, SOD) and administering a solution via a nasogastric tube containing topical, non-absorbable antibiotics such as gentamicin or tobramycin and colistin, plus an antifungal like nystatin. Additionally, a broad-spectrum intravenous antibiotic, commonly a third-generation cephalosporin, is administered during the first 3–4 days of ICU admission (Figure 1). This intervention is based on the premise that many microorganisms responsible for nosocomial infections in critically ill patients originate in the digestive tract. By reducing the bacterial load—eliminating potentially dangerous pathogenic bacteria such as certain Gram-negative species and fungi, while preserving non-pathogenic beneficial bacteria—the risk of infections can be significantly reduced [11].

Although SDD has shown efficacy in preventing infections in several studies [12,13], its application is not universal across ICUs due to several factors, including concerns about promoting antibiotic resistance, which could limit future treatment options [14]. Furthermore, the effectiveness of SDD varies depending on the environment and the specific characteristics of each ICU [15]. Ethical concerns about administering antibiotics to non-infected patients [16], additional costs, and the resources required for implementation and monitoring [17] also play a role, as well as the lack of consensus in international guidelines and recommendations [18], which leads to varying institutional policies and preferences [19].

In terms of antibiotic consumption, studies conducted in ICUs with SDD have yielded mixed results. Given these contradictory findings, it is essential to further investigate how the implementation of SDD actually impacts antibiotic consumption patterns. Some studies suggest that SDD reduces intravenous antibiotic use and prevents serious infections, which in the long term could help reduce selective pressure on bacteria. However, the prophylactic use of antibiotics at the digestive level raises concerns about the emergence of resistance in the gut microbiome [20].

Given the ongoing concerns about the impact of SDD on AMR, it is crucial to further explore the relationship between SDD implementation and the reduction in antibiotic consumption in ICUs. This requires continuous evaluation across ICUs in different regions, with detailed analyses of antibiotic consumption patterns over time. It is also important to consider the variety of resistant microorganisms and their responses to different antibiotic regimens in these clinical settings [21].

There are few large-scale studies that have specifically evaluated how SDD affects antibiotic consumption compared to other protocols. Most studies have primarily focused on clinical outcomes, such as mortality or the reduction in nosocomial infections in ICUs, while leaving aside comprehensive analyses of how antibiotic consumption patterns change before and after the implementation of SDD [22]

This study aims to demonstrate that implementing SDD in intensive care settings is associated with a significant decrease in antibiotic use. Such a reduction could help mitigate the selective pressure that drives the emergence and spread of multidrug-resistant bacteria, suggesting that SDD is not only an effective tool for infection prevention but also a key strategy in combating antimicrobial resistance.

## 2. Results

A total of 3266 (100%) patients were consecutively admitted during the study period. Of these, 1532 (46.91%) were admitted between June 2021 and June 2022, forming the pre-intervention control group. Subsequently, a total of 1734 (53.09%) patients were admitted between June 2022 and June 2023, forming the intervention group. The patients’ pre-ICU admission comorbidities, as recorded in the ENVIN study, were analyzed; however, no significant differences were found between the two study groups. The clinical characteristics of the patients are shown in Table 1.

### 2.1. Antibiotic Consumption

There was a significant decrease in overall antibiotic consumption in the post-SDD period compared to the pre-SDD period, with a total of 76.6 DDDs per 100 stays in the ICU in the pre-SDD group and 53.7 DDDs in the post-SDD group (Table 2) (mean differences = 1.04; 95% CI = 0.13–1.95; *p* = 0.028).

Based on therapeutic groups, a decrease in DDDs/100 stays in the post-SDD period was observed for carbapenems (Δ = −4.1), fourth-generation cephalosporins (Δ = −1.8), tetracyclines (Δ = −1.6), sulfonamides (Δ = −2.5), aminoglycosides (Δ = −0.6), monobactams (Δ = −0.4), and quinolones (Δ = −5.8). This translates into a reduction in the consumption of these antibiotics by 27.7%, 62.1%, 72.7%, 54.3%, 28.6%, 50.0%, and 49.3%, respectively. Individually, it is relevant to mention that the consumption of daptomycin decreased by 56.7% after the implementation of the SDD protocol in the ICU (Table 2).

Although there was a significant decrease in the consumption of most antibiotics, it is important to highlight that there were therapeutic groups that consumed more grams (g) of antibiotics in the post-SDD period than in the pre-SDD period, increasing their consumption by 14.0% for third-generation cephalosporins, 5.5% for macrolides, and 4.0% for β-lactams (Table 2).

### 2.2. Microbiological Findings

There was a decrease in the incidence density (ID) of colonizations, understood as the number of colonizations per 100 days of patients’ ICU stay, in the post-SDD period. The ID in the pre-SDD period was 3.26, while in the post-SDD period, it was 2.36, so the difference tended to be significant (IDR = 0.724; 95% CI = 0.467–1.117; *p* = 0.127).

**Figure 2:**
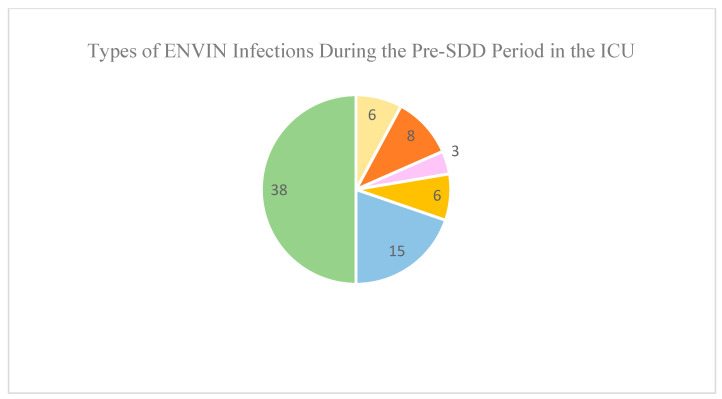

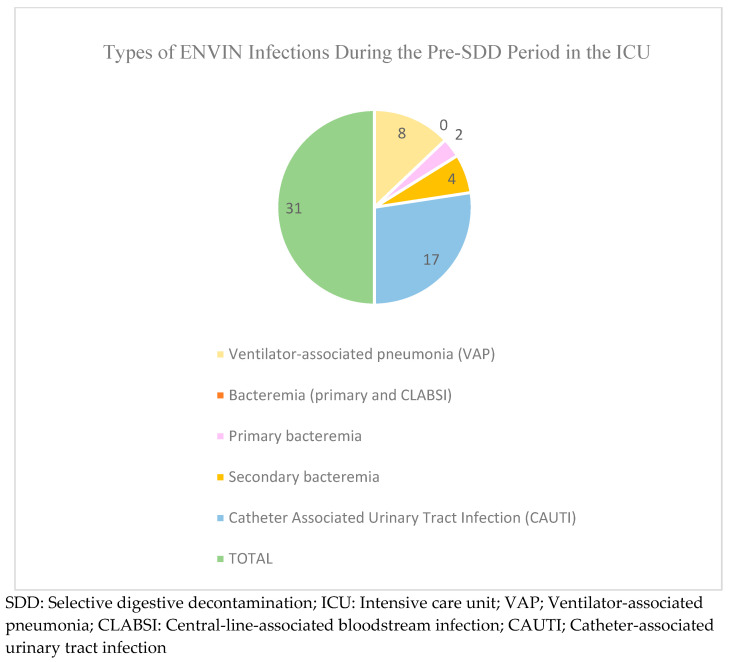
Types of ENVIN infections.

The number of nosocomial infections in the pre-SDD period was 38 (mechanical ventilation-associated pneumonia: six; primary or secondary bacteremia: 17; and catheter-associated urinary tract infection: 15) in 1531 patients admitted to the ICU; however, in the post-SDD period there were 31 infections (mechanical ventilation-associated pneumonia: eight; primary or secondary bacteremia: six; and catheter-associated urinary tract infection: 17) in 1734 patients. The ID of ENVIN infections in the pre-SDD period was 9.21, while in the post-SDD period, it was 6.54 (IDR = 0.711; 95% CI = 0.428–1.172; *p* = 0.16) (Figure 2).

On the other hand, we observed a decrease in the occurrence of carbapenemase-producing Enterobacterales (CPEs) in the post-SDD period compared to the pre-SDD period. The detection of CPE bacteria decreased from thirty-three to only seven, resulting in a significant decrease (mean differences = 2.31; CI 95% = 0.66–3.95; *p* = 0.0099). The isolated bacteria are shown in Table 3.

## 3. Discussion

The novelty of our study lies in the evaluation of the impact of implementing SDD within a specific intensive care unit and under a particular epidemiological context. While SDD is a well-established strategy, its application varies widely across different settings, and its potential effects on antimicrobial resistance make ongoing evaluation essential. Our study not only confirms the efficacy of SDD in reducing antibiotic consumption but also demonstrates a significant reduction in colonization rates by CPE. Furthermore, we emphasize that SDD has not been universally adopted, partly due to concerns about its potential to promote antibiotic resistance. However, given the inappropriate use of antibiotics and the challenge of developing new therapeutic strategies, MDR bacteria have become a major threat to global health [23]. Several meta-analyses have shown that the routine use of SDD does not increase the risk of developing MDR bacteria in patients with nosocomial infections. Additionally, SDD has been proven to be cost-effective, to prevent severe infections, and to reduce mortality risk in critically ill patients [4].

The inappropriate use of antibiotics and the challenge of developing new therapeutic strategies have made MDR bacteria a major threat to global health [23]. Several meta-analyses have shown that the routine use of SDD does not increase the risk of developing MDR bacteria in patients with nosocomial infections. Furthermore, SDD has been demonstrated to be cost-effective, to prevent severe infections, and to reduce the risk of mortality in critically ill patients [4].

In this regard, the ICU warrants special attention due to its high antimicrobial consumption. The elevated use of antibiotics in this setting may be attributed to the need for prompt and appropriate empirical treatment, which is crucial for managing critically ill patients. However, initial treatments are not always targeted due to a lack of diagnosis of the underlying infection and inadequate therapeutic de-escalation. Consequently, the indiscriminate use of antibiotics in the ICU, coupled with a lack of monitoring of potentially pathogenic colonizing microorganisms, promotes the spread of antibiotic resistance within these hospital areas.

On the other hand, the number of DDDs per 100 stays, defined as the time from a patient’s admission to the hospital until their discharge, whether they return home, transfer to another care center, or die, has proven to be a simple and effective method for monitoring antibiotic consumption following the implementation of measures to optimize antibiotic use, as corroborated by Collado et al., 2015 [24]. Additionally, DDDs per 100 stays can be used to evaluate the exposure of a given medical service to antimicrobials over a defined period, yielding promising results.

In our study, we detected that antibiotic consumption was reduced in the post-SDD period, with a significant decrease in carbapenems and quinolones. Additionally, a significant reduction in carbapenemase-producing bacteria (MDR bacteria) was observed during this period. It is known that the indiscriminate use of carbapenems has led to the emergence of bacteria with resistance mechanisms to these antibiotics, which is a global concern since carbapenems are often the last resort for treating MDR Gram-negative bacilli, specifically those producing AmpC and extended-spectrum beta-lactamases. The WHO has classified carbapenem-resistant Enterobacterales (CREs) as pathogens of critical priority in the search for new therapeutic alternatives, as they pose a significant challenge to universal health care [25]. Based on this, SDD appears to be an encouraging measure for preventing the emergence of MDR bacteria and colonizations, aligning with the results obtained by Sánchez-Ramirez et al., 2018 [11], which showed a significant reduction (*p* < 0.001) in infections caused by MDR bacteria (relative risk = 0.31; 95% CI, 0.23–0.41) after applying the SDD protocol and was associated with low rates of colistin- and tobramycin-resistant colonizing microorganisms [26].

In parallel with the implementation of SDD, a decrease in the use of daptomycin has also been observed, which seems to support the guidelines of the experts from the European Society for Clinical Microbiology and Infectious Diseases (EUCAST). These guidelines restrict this high-impact antibiotic to skin and soft tissue infections caused by Staphylococcus aureus and Streptococcus pyogenes, as well as to right-sided endocarditis caused by Staphylococcus aureus [27].

Regarding the use of quinolones, the decrease in the post-SDD period has been influenced by health alerts from the United States Food and Drug Administration (FDA) and the European Medicines Agency (EMA) related to the safety of these antibiotics. The FDA has reported severe hypoglycemia and significant psychological disturbances in patients treated with quinolones [28], while the EMA has associated fluoroquinolones with prolonged (lasting months or years), severe, disabling, and potentially irreversible adverse reactions affecting multiple systems, organ classes, and senses [29]. For all these reasons, the use of quinolones is increasingly restricted.

Another important aspect of our results is that the consumption of third-generation cephalosporins increased by 14% in the post-SDD period compared to the pre-SDD period. This observed trend underscores the importance of monitoring cephalosporin use in these clinical units to prevent the emergence of bacteria resistant to these antimicrobials. In our setting, the resistance rate to cephalosporins is less than 10%; however, in contexts with a high prevalence of resistance to these antibiotics, third-generation cephalosporins should be excluded from the SDD protocol.

**Figure 3:**
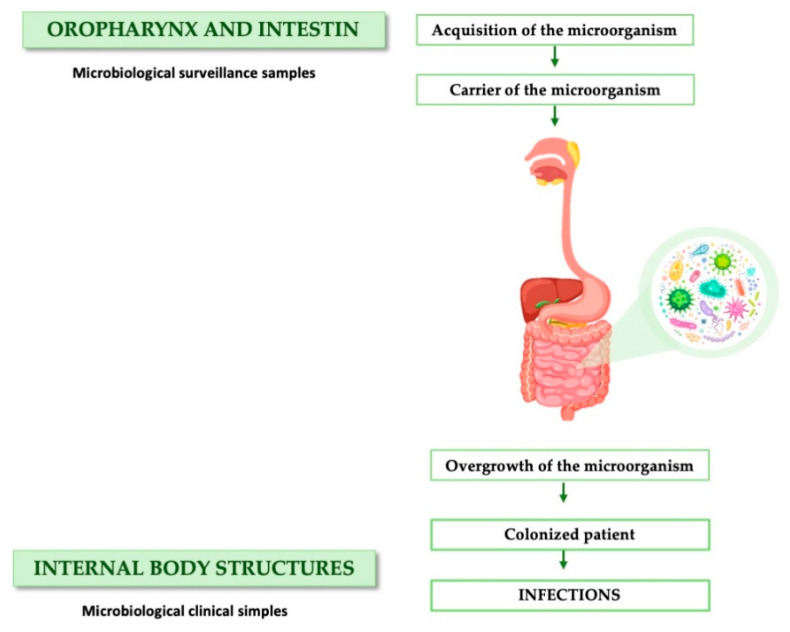
Pathogenesis of colonization and infection by potentially pathogenic microorganisms in critically ill patients.

Regarding the microbiological results obtained, it is important to highlight that most infections in the ICU are typically caused by the overgrowth of microorganisms in the intestine and oropharynx, which then migrate to other body structures and cause damage. Therefore, our findings are potentially beneficial for critically ill patients, as the SDD protocol not only reduces the incidence of nosocomial infections but also decreases the number of colonizing microorganisms (Figure 3).

Finally, although SDD has historically been viewed with caution due to the perceived risk of promoting antimicrobial resistance, our findings, along with the existing literature, suggest otherwise. Not only is SDD not associated with increased resistance, but it may also be an effective strategy to reduce the spread of MDR bacteria. This underscores the importance of considering SDD as part of a comprehensive approach to infection management in the ICU, especially in an environment in which antimicrobial resistance remains a significant threat.

### 3.1. Future Research Directions

One of the most underexplored aspects of SDD is its long-term effect on the intestinal microbiome. Investigating how SDD alters the microbial composition of the digestive tract is crucial to optimizing its use and minimizing potential adverse effects, such as bacterial imbalances or the development of antibiotic resistance. Understanding these dynamics could lead to more personalized SDD approaches, tailored to patients’ individual risk profiles, including comorbidities and microbiota composition [30].

Regarding the development of new antibiotic formulations, further research should focus on developing new formulations of non-absorbable antibiotics that could be more effective or have a reduced impact on the intestinal flora. In parallel, there is a need to advance technology for better monitoring the long-term effects of SDD on patients’ health. Such innovations could ensure that SDD remains a safe and effective tool in the ICU setting [30]. As mentioned earlier, specific studies are needed to evaluate the impact of SDD on antimicrobial resistance across various hospital environments and countries, especially in regions with moderate-to-high rates of bacterial resistance. These studies are critical for determining whether SDD remains an effective and safe intervention in the face of increasingly resistant bacteria [22].

Finally, regarding the implementation of the SDD protocol in underdeveloped countries, these countries face several economic and structural challenges. The upfront costs of non-absorbable antibiotics and solution preparation can be prohibitive, particularly in health systems with limited budgets. Additionally, ICUs in these countries often lack the necessary resources for treatment delivery and microbiological monitoring [5]. A shortage of trained personnel further hampers the proper implementation of complex protocols like SDD [24]. Lastly, the long-term sustainability of SDD is challenging due to insufficient ongoing funding and reliance on external support [22].

### 3.2. Limitations

Our study has several limitations that we must acknowledge. First, we did not design our protocol to assess the impact of individual interventions on outcomes. Our approach was epidemiological with the objective of studying the impact of DDD on annual antibiotic consumption.

Second, the study had a retrospective, nonrandomized design. Given the before/after design of the study, the results could be biased due to residual confounding factors that were not accounted for. However, the study was designed to address a clinical need and represents real-life data following the implementation of a clinical practice measure.

Third, our study was conducted at a single center, and the clinical results may not be directly translatable to other centers. Finally, other outcomes such as the duration or adequacy of antimicrobial treatment were not recorded. The aim of our study was to evaluate antibiotic consumption, specifically to compare the pattern of antibiotic consumption in our hospital’s ICU before and after the implementation of the SDD protocol.

Furthermore, we did not adjust the data to account for different prescribing patterns among physicians. However, in our hospital, prescribing patterns are guided by standardized protocols established by the Antibiotic Stewardship Program, which likely minimizes this influence. Nonetheless, it would be interesting to record the prescribing physician as a parameter, as we might still observe differences despite the use of these standardized protocols. For all these reasons, it would be interesting and it is necessary to conduct further studies comparing various groups while considering these limitations to obtain more conclusive results.

## 4. Materials and Methods

### 4.1. Study Design and Population

This is a pre/post quasi-experimental study involving an intervention. The intervention involved the implementation of the SDD protocol in the ICU of a second-level university hospital equipped with 22 beds. Two one-year periods were compared: the pre-SDD period in the ICU (June 2021–June 2022) and the post-SDD period in the ICU (June 2022–June 2023). The demographic variables collected from the patients included the following: sex, age, diagnosis on admission, patient origin (community or other hospital unit), patient exitus during ICU stay, length of ICU stay, and severity status measured using the APACHE-II (Acute Physiology and Chronic Health Evaluation II) scale. Also, patients’ pre-ICU admission comorbidities, as recorded in the ENVIN study, were analyzed, including diabetes, renal failure, immunosuppression, neoplasms, cirrhosis, COPD, malnutrition with hypoalbuminemia, and solid organ transplantation.

The SDD protocol was applied to patients who met the following criteria: positive growth in any clinical or surveillance sample of any MDR microorganism; patients who underwent tracheal intubation lasting more than 72 h; non-intubated patients who presented any risk factor (necrotizing pancreatitis, transplant and/or neutropenia, burns, or low level of consciousness with a Glasgow Coma Score ≤ 11/15 or a Glasgow Coma Score ≤ 8T/11).

Both the suspension (nasogastric tube) and the mouthpaste (SOD) were prepared by the hospital pharmacy service following the standards for correct preparation and quality control of master formulas. Both had the same composition, so that for each 125 mg (mouthpaste) or 125 mL (suspension), there were 2.5 g of colistin sulfate, 4 g of gentamicin sulfate, and 2.5 g of nystatin. The protocol was applied to the selected patients after adequate oral cleaning using a 0.1% chlorhexidine aqueous solution while aspirating secretions. It consisted of the following: (1) topical application to the buccal mucosa and oropharynx of 0.5 g of mouthpaste every six hours; (2) application of 20 milliliters of suspension every six hours via nasogastric tube or orally if the patient did not have a nasogastric tube; and (3) administration of a third-generation cephalosporin during their first four days of admission to the ICU, unless the patient had already been treated with specific antibiotics active against Gram-negative bacteria, in which case no additional antibiotics were administered.

In addition to the SDD protocol, critical patient safety programs (Zero Projects: zero bacteremia, zero pneumonia, and zero urinary tract infections), including proper catheter use, hand hygiene, and ventilator management, were also implemented. These interventions remained consistent throughout both study periods and were part of the infection prevention protocols in our intensive care unit.

### 4.2. Antimicrobial Consumption Data

The following are all the antibiotics routinely used in the ICU that were selected for this study: doxycycline (IV), ampicillin, amoxicillin-clavulanate (IV), piperacillin-tazobactam (IV), cefotaxime (IV), ceftazidime (IV), ceftriaxone (IV), cefepime (IV), aztreonam (IV), imipenem-cilastatin (IV), meropenem (IV), ertapenem (IV), sulfamethoxazole-trimethoprim (IV), azithromycin (IV), amikacin (IV), ciprofloxacin (IV), levofloxacin (IV), moxifloxacin (IV), vancomycin (IV), metronidazole (IV), fosfomycin (IV), linezolid (IV), and daptomycin (IV). The antibiotic consumption data were obtained through the ATHOS Pharmacy economic management module. For data extraction, an Excel database was designed to record the characteristics of antimicrobial consumption: type of antimicrobial and amount consumed in grams. Next, DDD per 100 stays was analyzed for each selected antibiotic using the following formula: DDD per 100 stays = (grams of selected antibiotic consumed/DDD in grams) × 100/number of stays in the chosen period, according to the ATC/DDD methodology based on the 2014 version of the WHO ATC/DDD classification [26].

### 4.3. Nosocomial Infection Prevention Measures

Nosocomial infection prevention measures were applied throughout the study (both periods) according to the established national protocols (Pneumonia Zero^®^, Bacteremia Zero^®^, and Resistance Zero^®^) in ENVIN-HELICS (vhebron.net) (accessed on 20 May 2024). As part of the clinical routine of the unit and following the recommendations of the Resistance Zero^®^ program, microbiological surveillance was performed to detect the colonization status of the patients. For this purpose, samples of the pharyngeal microbiota and perianal exudate were taken upon admission of all patients meeting the DSS criteria and then weekly throughout their stay in the unit.

### 4.4. Identification of Multidrug-Resistant Bacteria

The identification of ICU patients carrying MDR bacteria was conducted by collecting pharyngotonsillar, perianal, and nasal swabs every Tuesday. These swabs were sent to the clinical microbiology service to detect the presence of methicillin-resistant Staphylococcus aureus (MRSA) in the nasal samples, which were cultured using CHROMID^®^ MRSA SMART medium. Additionally, the presence of extended-spectrum β-lactamase (ESBL)-producing bacteria was evaluated by culturing using CHROMID^®^ ESBL medium, while carbapenemase production was assessed using CHROMID^®^ CARBA SMART medium in the pharyngotonsillar and perianal samples.

After 24 and 48 h, bacterial growth was assessed on the respective media. If growth was detected, the microorganism was identified using matrix-assisted laser desorption/ionization (MALDI).

To study the resistance of the isolated microorganisms, an antibiogram was performed, except when growth occurred on CHROMID^®^ CARBA SMART medium. In such cases, the presence of carbapenemases was investigated using immunochromatography (NG-Test^®^ CARBA-5).

Finally, when an ICU patient was identified as a carrier of a bacterium with antibiotic resistance mechanisms, both the critical care team and the hospital’s preventive medicine service were notified to proceed with the patient’s isolation.

### 4.5. Statistical Analysis

Differences between pre- and post-intervention cohorts for continuous variables were assessed using a 2-sample t-test or Wilcoxon rank-sum test, depending on the distribution. Differences between categorical variables were assessed using the χ^2^ test or Fisher’s exact test, as appropriate. To evaluate the change in the incidence density of infectious complications and colonizations, we used rate ratios (RRs), also known as incidence density ratios (IDRs). A *p*-value of <0.05 was considered statistically significant.

Finally, a comparative analysis was conducted on the data obtained from both periods to measure the efficacy in reducing antibiotic consumption with the implementation of SSD protocol in the ICU. The results were expressed descriptively as means, medians, and proportions according to the type of variable studied. The Epidat program was used.

### 4.6. Ethical Statement

The protocol of this study was approved by the Clinical Research Ethics Committee of Granada (CEIC) (approval ID 0600-N-23). All patients provided their consent before being included in the study.

## 5. Conclusions

SDD is emerging as a promising tool for reducing antibiotic consumption and preventing nosocomial infections without contributing to antimicrobial resistance. Given the severity of the antibiotic resistance crisis, SDD should be more widely considered for implementation in ICUs, thereby improving critical patient care and supporting the global fight against antimicrobial resistance.

## Figures and Tables

**Table 1:** Clinical characteristics of the patients included in the study.

	Pre-SDD Period (n = 1532)	Post-SDD Period (n = 1734)	*p* Value
Sex (M/F) (%)	67.4/32.6	65.3/34.3	0.38
Age (years); (Mean ± SD)	61.5 ± 16.1	63.1 ± 15.5	0.14
APACHE II; (Mean ± SD)	14.7 ± 9.4	15.7 ± 9.8	0.44
SOFA admission; (Mean ± SD)	6.7 ± 3.2	6.3 ± 4.1	0.81
Admission diagnosis (%)			0.75
Cardiological	41.3	40.5	
Medical	38.9	41.5	
Surgical	16.1	14.3	
Trauma	3.7	3.7	
Length of MV; (Median, IQR)	4.1 (2–8)	4.5 (2–9)	0.45
Length of ICU stay; (Median, IQR)	4.5 (2–9)	4.1 (2–8)	0.28
Mortality rate (%)	10.4	12.6	0.08

SDD: Selective digestive decontamination; M: male; F: Female; APACHE: Acute Physiology and Chronic Health Evaluation; SOFA: Sequential Organ Failure Assessment; MV: Mechanical ventilation; ICU: Intensive care unit; IQR: Interquartile range.

**Table 2:** Comparison of DDDs per 100 stays in the pre-implementation period of SDD (pre-SDD) in ICU (June 2021–June 2022) and the post-implementation period of SDD (post-SDD) in ICU (June 2022–June 2023).

	Consumption (g) Pre-SDD Period	Consumption (g) Post-SDD Period	DDD/100 Stays Pre-SDD in ICU	DDD/100 Stays Post-SDD in ICU	Δ
Doxycycline	224	64	2.2	0.6	−1.60
**Tetracyclines**	**224**	**64**	**2.2**	**0.6**	**−1.60**
Ampicillin	1051	707	1.8	1.0	−0.80
Amoxicillin	1345	1443	5.2	5.6	0.40
Piperacillin	1023	1510	2.9	3.7	0.80
**β-lactams (Penicillins)**	**3419**	**3660**	**9.9**	**10.3**	**0.40**
Ceftazidime	619	275	1.6	0.6	−1.0
Cefotaxime	352	511	0.9	1.1	0.20
Ceftriaxone	867	1092	1.8	3.3	1.50
**Third-Generation Cephalosporins**	**1838**	**1878**	**4.3**	**5.0**	**0.70**
Cefepime	581	248	2.9	1.1	−1.80
**Fourth-Generation Cephalosporins**	**581**	**248**	**2.9**	**1.1**	**−1.80**
Aztreonam	304	204	0.8	0.4	−0.40
**Monobactams**	**304**	**204**	**0.8**	**0.4**	**−0.40**
Meropenem	3433	2754	11.5	8.0	−3.50
Ertapenem	229	228	2.3	2.0	−0.30
Imipenem	418	346	1.0	0.7	−0.30
**Carbapenems**	**4080**	**3228**	**14.8**	**10.7**	**−4.10**
Sulfamethoxazole/Trimethoprim	1727	900	4.6	2.1	−2.50
**Sulfonamides**	**1727**	**900**	**4.6**	**2.1**	**−2.50**
Azithromycin	182	223	1.8	1.9	0.10
**Macrolides**	**182**	**223**	**1.8**	**1.9**	**0.10**
Amikacin	412	356	2.1	1.5	−0.60
**Aminoglycosides**	**412**	**356**	**2.1**	**1.5**	**−0.60**
Ciprofloxacin	213	325	0.5	0.7	0.20
Levofloxacin	1229	736	12.3	6.4	−5.90
Moxifloxacin	63	53	0.6	0.5	−0.10
**Quinolones**	**1505**	**1114**	**13.4**	**7.6**	**−5.80**
Vancomycin	251	61	0.9	0.2	−0.70
Metronidazole	480	345	1.5	1.0	−0.50
Linezolid	1111	1482	5.6	6.4	0.80
Daptomycin	717	352	12.0	5.2	−6.80
**Other Antibacterials**	**2559**	**2240**	**20.0**	**12.8**	**−7.20**
**TOTAL**	**16,973**	**14,153**	**76.6**	**53.7**	**−22.00**

**Table 3:** MDR bacteria isolated during the pre-SDD and post-SDD periods.

Carbapenemases	Pre-SDD Period in ICU	Post-SDD Period in ICU
KPC-producing *Keblsiella pneumoniae*	1	0
OXA-48 and ESBL-producing *Escherichia coli*	3	0
OXA-48-producing *Escherichia coli*	4	1
OXA-48 and ESBL-producing *Enterobacter cloacae complex*	1	0
OXA-48-producing *Enterobacter cloacae complex*	9	0
OXA-48 and ESBL-producing *Klebsiella pneumoniae*	1	2
OXA-48-producing *Klebsiella pneumoniae*	3	1
OXA-48-producing *Citrobacter freundii*	1	2
OXA-48-producing *Serratia marcescens*	1	0
VIM-producing *Enterobacter cloacae complex*	3	0
VIM- and OXA-48-producing *Enterobacter cloacae complex*	7	1
VIM-producing *Pseudomonas aeruginosa*	2	0
IMP-producing *Pseudomonas aeruginosa*	1	0
TOTAL	37	7

SDD: Selective digestive decontamination; ICU: Intensive care unit; KPC: *Klebsiella Pneumoniae Carbapenemase*; OXA-48: *Oxacilinase-48*; ESBL: *Extended Spectrum β-lactamases*; VIM: *Verona Integron-encoded Metallo-betalactamase*; IMP: *Active On Imipenem*.

## Data Availability

The data presented in this study are available upon request from the corresponding author. The data are not publicly available as they contain clinical and personal information.
